# Clinical pitfalls in the diagnosis of segmental overgrowth syndromes: a child with the c.2740G > A mutation in *PIK3CA* gene

**DOI:** 10.1186/s13052-018-0568-8

**Published:** 2018-09-19

**Authors:** Alice Maguolo, Franco Antoniazzi, Alice Spano, Elena Fiorini, Rossella Gaudino, Margherita Mauro, Gaetano Cantalupo, Paolo Biban, Silvia Maitz, Paolo Cavarzere

**Affiliations:** 1 Pediatric Division, Department of Pediatrics, University Hospital of Verona, Verona, Italy; 2 Regional Center for the Diagnosis and Treatment of Children and Adolescents Rare Skeletal Disorders, Pediatric Clinic, Department of Surgical Sciences, Dentistry, Gynecology and Pediatrics, University of Verona, Verona, Italy; 3 Medical Genetic Specialization, University of Milan, Milan, Italy; 4 Child Neuropsychiatry, Department of Surgical Sciences, Dentistry, Gynecology and Pediatrics, University of Verona, Verona, Italy; 5 Pediatric Intensive Care Unit, Universitary Hospital of Verona, Verona, Italy; 6 Clinical Pediatric Genetics Unit, Pediatrics Clinics, MBBM Foundation, S. Gerardo Hospital, Monza, Italy

**Keywords:** Overgrowth, Hemihyperplasia, *PIK3CA* gene, Megalencephaly-capillary malformation syndrome

## Abstract

**Background:**

Overgrowth syndromes are known as a heterogeneous group of conditions characterized by a generalized or segmental, symmetric or asymmetric, overgrowth that may involve several tissues. These disorders, which present a wide range of phenotypic variability, are often caused by mosaic somatic mutations in the genes associated with the PI3K/AKT/mTOR cellular pathway, a signaling cascade that plays a key role in cellular growth. Overgrowth syndromes are frequently misdiagnosed. Given that they are also associated to an increased oncologic risk, it is important to distinguish the clinical characteristic of these disorders since the first months of life.

**Case presentation:**

We report the case of a seven-year-old male child with macrocephaly and right lateralized overgrowth, reported from birth. The patient arrived to our attention after an initial diagnosis of isolated benign macrocephaly was formulated at the age of 12 months. Afterwards, the child presented a moderate intellectual disability and pain episodes at right lower limb. We repeated a brain Magnetic Resonance Imaging that revealed ventriculomegaly, cerebellar tonsillar ectopia, a markedly thick corpus callosum, and white matter abnormalities. The diagnosis of segmental overgrowth syndrome was formulated according to the clinical presentation and confirmed by the finding of the variant c.2740G > A in the gene *PIK3CA* presented in somatic mosaicism.

**Conclusions:**

Our patient is the first children with the c.2740G > A variant in *PIK3CA* gene reported in Italy. We underline the importance of the genotype-phenotype correlation in the diagnostic process of overgrowth syndromes and emphasize the strict correlation between the mutation c.2740G > A in the *PIK3CA* gene and the Megalencephaly-Capillary Malformation syndrome phenotype.

## Background

Overgrowth syndromes (OSs) are known as a heterogeneous group of conditions characterized by a generalized or segmental, symmetric or asymmetric, overgrowth that may involve many tissues such as bones, muscles, adipose tissue, skin and nerves. These disorders may manifest at birth or develop in the early childhood and can be associated to an increased oncologic risk [1]. The more frequent anomalies associated with segmental OSs are hamartomas, epidermal nevi, lymphovascular malformations, lipomatosis, hemihypertrophy/hemihyperplasia (these terms have been recently replaced with “lateralized overgrowth” [2]), polydactyly, encephalic developmental anomalies, macrocephaly and other skull abnormalities [1].

In the majority of cases, segmental OSs are caused by mosaic somatic mutations in the genes associated with the PI3K/AKT/mTOR cellular pathway, a signaling cascade that plays a key role in cellular growth [3]. In mosaic diseases, the mutations occur during mitotic cell division, producing two or more genetically distinct cell lineages originating from a single zygote [4]. The wide range of phenotypic variability of segmental OSs and their overlapping features may be explained by the timing of the mutation’s occurrence during the embryonic development, the tissue localization of the mutation, the level of mosaicism and the potential allelic heterogeneity [5].

## Clinical CASE

A seven-and-a-half-year-old male child was sent to our Pediatric Endocrinology Centre for macrocephaly and right lateralized overgrowth, reported from birth. Parents were not related and no noteworthy diseases were reported in his family history. The pregnancy was characterized by spontaneous abortion of the dizygotic twin at 16 gestational weeks. Fetal ultrasounds were normal. He was born at 35 weeks of gestational age by an emergency caesarean section for acute fetal suffering. Birth weight was 3010 g (1.65 standard deviations [SD]), birth length was 51 cm (2.45 SD) and birth occipito-frontal circumference (OFC) was 36 cm (2.93 SD).

The perinatal period was characterized by hospitalization because of the mild prematurity, neonatal jaundice treated with phototherapy and the findings of hypotonia. In his first months of life he presented a progressive increase of the OFC and was submitted to brain Magnetic Resonance Imaging (MRI) and to neurosurgical evaluation, which permitted an initial diagnosis of isolated benign macrocephaly. The MRI was repeated at the age of 2, revealing ventriculomegaly, Chiari Malformation type I and an arachnoid left temporo-polar cyst. At neurological evaluation, he presented a developmental delay characterized by an acquisition of sitting position at 30 months of life and autonomous walking at 3 years of life and a speech delay with first production of words after 2 years of age. Since he was 3 years old he has been suffering from pain episodes at right lower limb unrelated to physical activity or other specific events, usually characterized by prolonged duration, good response to paracetamol and associated to limb failure and fall to the ground.

At our first physical examination his weight was 24.9 kg (0.54 SD), height 118.3 cm (− 1.42 SD) and OFC 60.5 cm (> 3 SD). He had several capillary malformations on medial axis. His face presented two frontonasal hemangiomas, a hyperpigmented brownish stain on the forehead with telangiectasia, and two on flat hemangiomas the trunk; furthermore, the patient had low-set ears, teeth with serrated edges, diffuse muscular hypotonia, joint hypermobility, and a fine and gross motor dyspraxia associated to a mild intellectual disability. His right lateralized overgrowth involved face, trunk and limbs (mainly legs). In particular, he showed a mild asymmetry of the face and of the facial mime with the right side more represented, different length and diameter of the legs (the right were 66 cm and 40 cm respectively and the left one 63 cm and 36 cm) and of the forearms, measured from elbow to the end of the middle finger (the right were 29 cm and 14 cm respectively, the left one 26 cm and 12 cm).

X-ray, ultrasounds and MRI of lower limbs were performed confirming the asymmetry in length of the legs and showing a musculature and a panniculus adiposus of the right side more represented than the contralateral. Echocardiogram and abdominal ultrasound were normal.

**Fig. 1:**
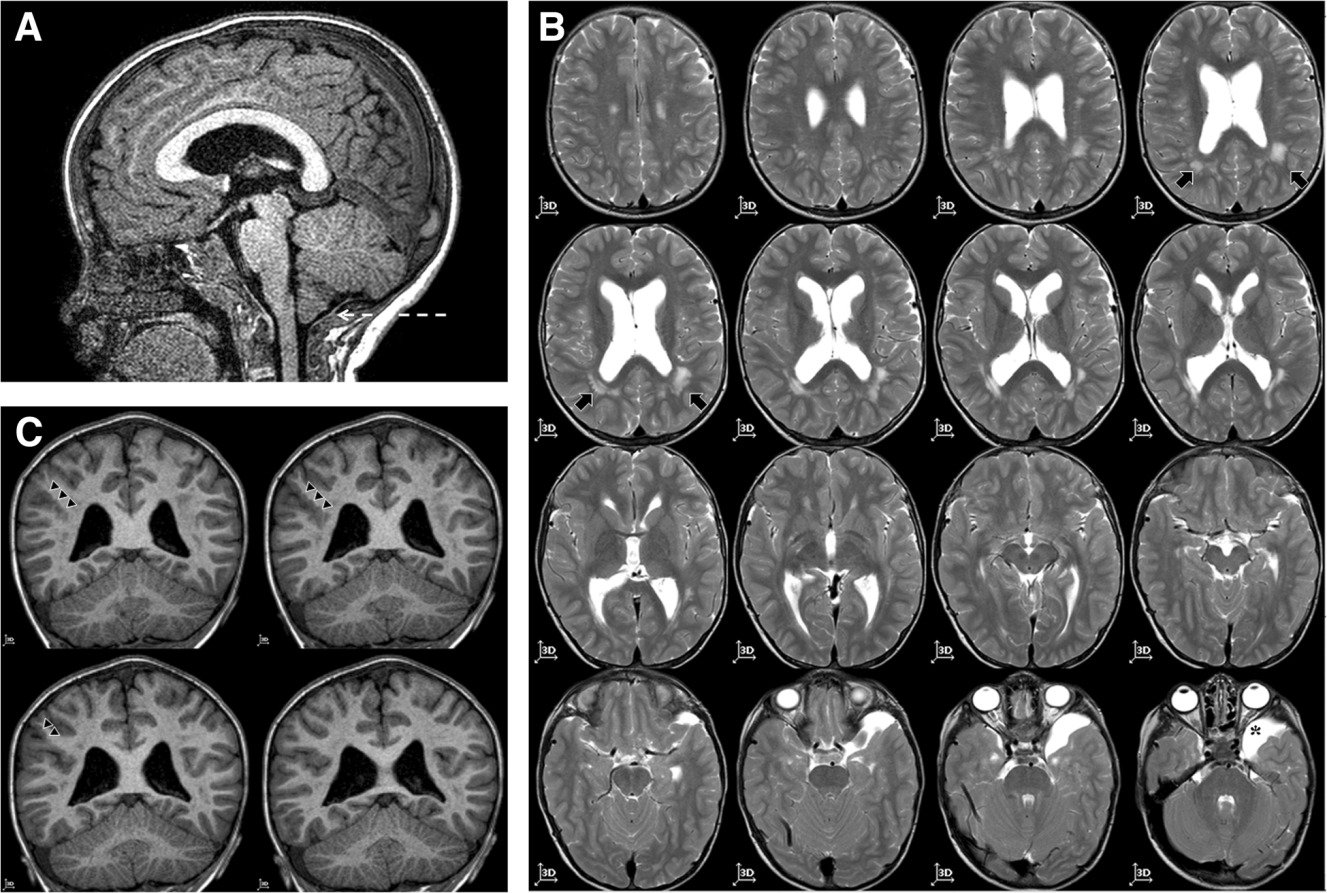
Brain MRI: markedly thick corpus callosum and Chiari malformation type I (highlighted by the white arrow) represented in panel **a**; abnormalities of white matter (highlighted by the black arrow) and arachnoid left temporo-polar cyst (highlighted by the asterisk) represented in panel **b**; area of polymicrogyria in right parietal region (highlighted by the black triangles) represented in panel **c**

Brain MRI was repeated confirming previous findings of ventriculomegaly, arachnoid left temporo-polar cyst, cerebellar tonsillar ectopia. Furthermore, it revealed a markedly thick corpus callosum (mega-CC), abnormalities of white matter, an area of polymicrogyria, and a pituitary gland with a mild reduction in volume for age (Fig. 1). The electroencephalography showed sporadic and isolated paroxysmal abnormalities. The functional evaluation permitted a diagnosis of mild intellectual disability, attention-deficit, hyperactivity disorder and emotional disturbance (Wechsler Intelligence Scale for Children-IV: Total Intelligence Quotient 51).

On the basis of these clinical signs and symptoms we hypothesized an OS and sent the patient to geneticists for specific evaluation. Pediatric geneticists of the Pediatric Department of MBBM Fundation, Monza, Italy, confirmed our diagnostic suspicion and diagnosed a segmental OS. Consequently, the child was submitted to the molecular analysis of 21 selected genes involved in the PI3K/AKT/mTOR pathway (*PIK3R1, PIK3R2, PIK3CA, PTEN, PDK1, PDK2, KRAS, AKT1, AKT2, AKT3, RICTOR, MAPKAP1, MLST8, MTOR, IRS1, GAB1, GAB2, THEM4, MAPK8IP1, PTPN11, RPTOR*). To identify causative mosaic somatic mutation on these genes the genomic DeoxyriboNucleic Acid (DNA) was extracted from cutaneous biopsy of affected tissue and Targeted Next generation sequencing (NGS) was performed. The pathogenic point mutation c.2740G > A (pGly914Arg) in *PIK3CA* exon 18 was isolated in the genomic DNA of our patient. It was identified in heterozygosity and was presented as somatic mosaic with a frequency of 31.2%. The identified variant was verified by Sanger sequencing.

## Discussion and conclusions

Our patient is the first child with the c.2740G > A (pGly914Arg) variant in *PIK3CA* gene reported in Italy and the 19th case described in literature. The detection of this somatic mutation confirmed our clinical suspicion of segmental OS.

*PIK3CA* gene, localized on chromosome 3 in the q26.32 position, encodes for the catalytic subunit p110α of PI3K. When growth factors stimulate PI3K, its catalytic subunit p110α converts phosphatidylinositol 4,5-bisphosphate (PIP2) to phosphatidylinositol (3,4,5)-trisphosphate (PIP3) and permits activation of AKT protein and then the activation of mTOR signalization pathway that induces cellular proliferation [3]. Available functional studies have demonstrated that several variants in p110α disrupt the inactive conformation of PIK3 complex and maintain the catalytic subunit in a high activity state [6].

Recently, segmental OSs caused by somatic activating mutation of the gene *PIK3CA* were unified and collected under the term of “*PIK3CA*-Related Overgrowth Spectrum (PROS)”, stressing the importance of the genotype-phenotype correlation analysis in the diagnostic process of these diseases [3] (Table 1). In particular, the c.2740G > A variant found in our patient was already identified and was considered pathogenic in 18 patients with Megalencephaly-Capillary Malformation-Polymicrogyria syndrome (MCAP) [7–11]. MCAP was first described in 1997 [12, 13] and is phenotypically characterized by involvement of the Central Nervous System (CNS) and vascular anomalies [14]. In further detail, the phenotype of MCAP is characterized by a congenital or early postnatal megalencephaly, ventriculomegaly (that may lead to hydrocephalus), progressive cerebellar tonsillar ectopia leading to Chiari Malformation, and cortical brain abnormalities, such as polymicrogyria and white matter abnormalities [15]. Secondary neurological symptoms, such as developmental delay, ranging from mild to severe, hypotonia (especially neonatal onset), seizures, autistic features, and behavioral problems, such as unexplained irritability, attention deficit, hyperactivity disorder, and obsessive compulsive disorder, are also included in clinical presentation [15, 16]. Other clinical features associated to this syndrome are vascular anomalies, especially capillary malformations of midline face and body, distal limb anomalies, syndactyly and polydactyly, connective tissue dysplasia at variable degrees (skin hyperelasticity, skin laxity, joint hypermobility, and thick subcutaneous tissue), and mild focal or segmental somatic body overgrowth [16].

**Table 1:** PROS: *PIK3CA*-Related Overgrowth Spectrum

MCAP (megalencephaly-capillary malformations syndrome)	
DMEG (dysplastic megalencephaly)	
CLOVES (Congenital Lipomatous Overgrowth, Vascular Malformations, Epidermal Nevi, Scoliosis/Skeletal and Spinal syndrome)	
HHML (hemihyperplasia-multiple lipomatosis)	
Fibroadipose hyperplasia or Overgrowth (FAO)	
Klippel-Trenaunay Syndrome	
Fibroadipose Infiltrating Lipomatosis, Seborrheic keratosis (SK), Benign lichenoid keratosis (BLK), Epidermal nevi (EN)	

The diagnosis of MCAP syndrome could be established in our patient according to the clinical criteria of MCAP (Table 2), supported by the correlation between MCAP phenotype and the point mutation c.2740G > A in *PIK3CA* gene, already reported in literature [16]. Our patient, in fact, presented megalencephaly, Chiari malformation, ventriculomegaly, and white matter abnormalities at brain MRI, capillary anomalies, developmental delay, and right lateralized overgrowth. It is essential to emphasize that lateralized overgrowth is a peculiar clinical sign of the disease; however, it was not taken into consideration until pediatric endocrinological evaluation at the age of 7. Although there are no widely accepted criteria for defining lateralized overgrowth as distinct from normal growth variation in children [2], it is important to differentiate a paraphysiological asymmetry in the body and a lateralized overgrowth, as present in our patient, in order to obtain a definitive diagnosis as soon as possible.

**Table 2:** Diagnostic Criteria for MCAP: core feature (1) plus either two or three core features

Core features	Supportive features	Secondary features
(1) Early overgrowth (brain > somatic tissue) progressive megalencephaly	Selective brain overgrowth (ventriculomegaly, cerebellar tonsillar ectopia, abnormally thick cospus callosum); congenital somatic overgrowth, somatic or cranial asymmetry	Hypotonia Developmental delay Seizures
(2) Developmental vascular disorders capillary malformations (midline face and body)	Infantile hemangiomas, venous aneurysms, aberrant vasculature	
(3) Distal limb anomalies (syndactily)	Polydactyly, Sandal-gap toes	
(4) Cortical brain malformations (polymicrogyria)		
(5) Connective tissue dysplasia (skin hyperelasticity, joint ipermobility, thick doughy subcutaneous tissue)		

In PROS, an increase in the carcinogenic risk is also described. Somatic mutations in *PIK3CA* are very similar to those seen in many cancers, including glioblastoma and colorectal, ovarian, breast, and hepatocellular carcinomas [17]. Although malignancies have been reported with inferior recurrence in MCAP than in patients with other OSs, Wilms tumor, meningioma, and leukemia have been reported in MCAP patients [16]. Only the results of prospective studies will clarify the oncogenic risk of these disorders. Meanwhile, it would be prudent to recommend serial abdominal ultrasound every 3–4 months until the age of 8 years and neurological monitoring and spinal MRI scan in patients with truncal involvement, in order to exclude the presence of major nerve neurofibromas or vascular/lipomatous lesions [17]. Our patient did not present a malignancy in early childhood, even if he was not submitted to an adequate surveillance because of the delayed diagnosis. Although all these signs and symptoms may be correlated to MCAP, the diagnosis was achieved, in fact, only at the age of 8 years old. Initially the Chiari Malformation was misinterpreted as benign macrocephaly and analogously, other typical features of the syndrome identified at brain MRI, such as alteration of periventricular white matter, were considered at first evaluation as a consequence of neonatal suffering.

In some cases of segmental OSs clinical diagnosis might be difficult because the phenotypes have variable expression and overlapping features, due to the mosaicism of the mutation. In fact, only few patients present all the characteristic features described above [18]. In particular, our patient presented as initial main phenotypic characteristic an isolated macrocephaly that needed MRI and neurosurgical evaluation. This clinical sign might be associated with different OSs related to mutations of the PI3K/AKT/mTOR molecular pathway besides MCAP, such as megalencephaly-polymicrogyria-polydactyly-hydrocephalus syndrome (MPPH) [11], and PTEN related diseases, such as Cowden disease, Bannayan syndrome, and Proteus syndrome [19], that may be associated with different degree of developmental delay. This is precisely why it is necessary to have molecular diagnosis. Targeted next generation is the preferred method for molecular diagnosis of segmental OSs because it offers a much deeper sequencing coverage and allows the detection of low-level mosaicism [8]. In our case the genomic DNA was extracted from fibroblasts obtained from a biopsy of cutaneous affected tissue, usually considered the best sample for the molecular diagnosis of segmental OSs [16], when suspecting a mosaic somatic mutation. In fact, the mosaicism levels detected in blood samples are considerably lower compared to those seen in affected tissues [11].

For affected patients and their countless clinical problems the therapeutic approach must be as wide and multidisciplinary as possible. A specific treatment does not exist yet and until recently surgical debulking, orthopedic procedures, and vascular interventional techniques have been the only treatments available for patients with segmental OSs [19]. Nevertheless, in more recent years many clinical trials have been initiated utilizing small molecule inhibitors of the PI3K signaling network, previously studied in oncology drug development area [20]. The results of these studies appear promising and underline once again the value of the molecular analysis for a proper diagnosis.

In conclusion, we underline the strict correlation between MCAP phenotype and the pathogenic mutation c.2740G > A in the *PIK3CA* gene. Therefore, we recommend physicians to suspect a segmental OS in general, and a MCAP in particular, in presence of generalized or segmental overgrowth, overall if associated to megalencephaly and other brain MRI anomalies, developmental delay, vascular malformations and connective tissue dysplasia. In presence of these clinical signs and symptoms it is mandatory for a pediatrician to send the patient for a genetic evaluation at an early stage. In relation to genetic indication, they should moreover submit the patient to a molecular analysis in order to reach the appropriate diagnosis and to establish the more correct follow up and treatment.

## Abbreviations

CNS: Central Nervous System
DNA: DeoxyriboNucleic Acid
MCAP: Megalencephaly-Capillary Malformation-Polymicrogyria syndrome
MRI: Magnetic Resonance Imaging
NGS: Next Generation Sequencing
OFC: Occipito-frontal circumference
OSs: Overgrowth syndromes
PIP2: To phosphatidylinositol 4,5-bisphosphate
PIP3: Phosphatidylinositol (3,4,5)-trisphosphate
PROS: *PIK3CA*-Related Overgrowth Spectrum
SD: Standard deviations
